# Putative Iron-Sulfur Proteins Are Required for Hydrogen Consumption and Enhance Survival of Mycobacteria

**DOI:** 10.3389/fmicb.2019.02749

**Published:** 2019-11-22

**Authors:** Zahra F. Islam, Paul R. F. Cordero, Chris Greening

**Affiliations:** School of Biological Sciences, Monash University, Clayton, VIC, Australia

**Keywords:** hydrogenase, *Mycobacterium*, atmospheric H_2_, iron-sulfur protein, hydrogen cycle

## Abstract

Aerobic soil bacteria persist by scavenging molecular hydrogen (H_2_) from the atmosphere. This key process is the primary sink in the biogeochemical hydrogen cycle and supports the productivity of oligotrophic ecosystems. In *Mycobacterium smegmatis*, atmospheric H_2_ oxidation is catalyzed by two phylogenetically distinct [NiFe]-hydrogenases, Huc (group 2a) and Hhy (group 1h). However, it is currently unresolved how these enzymes transfer electrons derived from H_2_ oxidation into the aerobic respiratory chain. In this work, we used genetic approaches to confirm that two putative iron-sulfur cluster proteins encoded on the hydrogenase structural operons, HucE and HhyE, are required for H_2_ consumption in *M. smegmatis*. Sequence analysis show that these proteins, while homologous, fall into distinct phylogenetic clades and have distinct metal-binding motifs. H_2_ oxidation was reduced when the genes encoding these proteins were deleted individually and was eliminated when they were deleted in combination. In turn, the growth yield and long-term survival of these deletion strains was modestly but significantly reduced compared to the parent strain. In both biochemical and phenotypic assays, the mutant strains lacking the putative iron-sulfur proteins phenocopied those of hydrogenase structural subunit mutants. We hypothesize that these proteins mediate electron transfer between the catalytic subunits of the hydrogenases and the menaquinone pool of the *M. smegmatis* respiratory chain; however, other roles (e.g., in maturation) are also plausible and further work is required to resolve their role. The conserved nature of these proteins within most Hhy- or Huc-encoding organisms suggests that these proteins are important determinants of atmospheric H_2_ oxidation.

## Introduction

Over the last decade, various studies have revealed that aerobic bacteria conserve energy during persistence through aerobic respiration of atmospheric hydrogen (H_2_) (Constant et al., 2010; Greening et al., 2014b, 2015a; Meredith et al., 2014; Liot and Constant, 2016; Islam et al., 2019). This process is now recognized to be important for biogeochemical and ecological reasons. Gas-scavenging soil bacteria serve as the primary sink in the global hydrogen cycle and are responsible for the net consumption of approximately 70 million tonnes of H_2_ each year (Constant et al., 2009; Ehhalt and Rohrer, 2009; Greening et al., 2014a; Piché-Choquette et al., 2018). More recently, it has been inferred that this process supports the productivity and biodiversity of various ecosystems, especially low-carbon soils (Lynch et al., 2014; Kanno et al., 2015; Khdhiri et al., 2015; Greening et al., 2016; Ji et al., 2017; Bay et al., 2018; Kessler et al., 2019; Piché-Choquette and Constant, 2019). Atmospheric H_2_ oxidation appears to be a widespread trait among soil bacteria. To date, bacteria from three phyla have been experimentally shown to oxidize atmospheric H_2_, Actinobacteriota (Constant et al., 2008, 2010; Greening et al., 2014a; Meredith et al., 2014), Acidobacteriota (Greening et al., 2015a; Myers and King, 2016), and Chloroflexota (Islam et al., 2019). However, genomic and metagenomic studies have indicated at least 13 other phyla encode enzymes that can mediate this process (Greening et al., 2016; Carere et al., 2017; Ji et al., 2017; Piché-Choquette et al., 2017).

The genetic basis and physiological role of atmospheric H_2_ oxidation is now largely understood. This process has been most comprehensively studied in the genetically tractable soil actinobacterium *Mycobacterium smegmatis* (Greening and Cook, 2014). In this organism, atmospheric H_2_ oxidation is mediated by two membrane-bound, oxygen-tolerant hydrogenases, Huc (group 2a [NiFe]-hydrogenase, also known as Hyd1 or cyanobacterial-type uptake hydrogenase) and Hhy (group 1h [NiFe]-hydrogenase, also known as Hyd2 or actinobacterial-type uptake hydrogenase) (Berney et al., 2014b). Additionally, *M. smegmatis* encodes a third [NiFe]-hydrogenase, Hyh (Hyd3), which mediates fermentative H_2_ production during hypoxia (Berney et al., 2014a). Both H_2_-oxidizing hydrogenases contain a large subunit containing the [NiFe] active site (HucL, HhyL) and a small subunit containing three iron-sulfur clusters (HucS, HhyS), as well as potential additional subunits (Berney et al., 2014b; Cordero et al., 2019b). These two hydrogenases are upregulated in stationary-phase cells, including in response to organic carbon limitation (Berney and Cook, 2010; Berney et al., 2014b). Consistently, when the structural subunits of these hydrogenases are deleted, strains show reduced growth yield and impaired long-term survival during starvation (Berney and Cook, 2010; Berney et al., 2014a; Greening et al., 2014b). Similar findings have been made in *Streptomyces avermitilis*; the sole hydrogenase of this organism, Hhy, is exclusively expressed in exospores and strains lacking this enzyme exhibit severe survival defects (Constant et al., 2010; Liot and Constant, 2016). Given these findings, it is proposed that bacteria shift from growing on organic compounds to persisting on atmospheric trace gases. Indeed, theoretical calculations indicate that the energy derived from atmospheric H_2_ oxidation (0.53 ppmv) can sustain the maintenance of 10^7^ to 10^8^ cells per gram of soil (Conrad, 1999).

Despite this progress, little is currently known about the biochemical basis of atmospheric H_2_ oxidation. One outstanding question is how electrons derived from H_2_ oxidation are transferred to the respiratory chain. Most classes of respiratory uptake hydrogenases are predicted to be co-transcribed with a cytochrome *b* subunit (Greening et al., 2016; Søndergaard et al., 2016). For example, such subunits interact with the prototypical oxygen-tolerant hydrogenases (group 1d [NiFe]-hydrogenases) of *Escherichia coli* and *Ralstonia eutropha*; they anchor the hydrogenase to the membrane and transfer electrons from the hydrogenase small subunit to the quinone pool (Frielingsdorf et al., 2011; Volbeda et al., 2013). However, we did not detect equivalent proteins in the operons encoding the structural subunits of Huc (MSMEG_2261–2270) or Hhy (MSMEG_2722 – 2718) in *M. smegmatis* (Supplementary Figure S1) (Berney et al., 2014b). Putative iron-sulfur proteins, tentatively annotated as HucE (MSMEG_2268) and HhyE (MSMEG_2718), were encoded downstream of the hydrogenase structural subunits and may potentially fulfill this role instead (Berney et al., 2014b; Greening et al., 2015b). In this work, we characterized the effects of deleting these genes on hydrogenase activity, growth, and survival in *M. smegmatis*. We also investigated their broader conservation in hydrogenase-encoding bacteria.

## Materials and Methods

### Bacterial Strains and Growth Conditions

All bacterial strains and plasmids used in this study are listed in Supplementary Table S1. *Escherichia coli* TOP10 was maintained on lysogeny broth (LB) agar plates (10 g L^–1^ tryptone, 5 g L^–1^ NaCl, 5 g L^–1^ yeast extract, 15 g L^–1^ agar), while *Mycobacterium smegmatis* mc^2^155 (Snapper et al., 1990) and derived mutants were maintained on LB agar plates supplemented with 0.05% (w/v) Tween 80 (LBT). For broth culture, *E. coli* was grown in LB. *M. smegmatis* was grown in either LBT or in Hartmans de Bont (HdB) minimal medium (Hartmans and De Bont, 1992) supplemented with 0.2% (w/v) glycerol. In all cases, liquid cultures were grown in rotary incubators at 37°C with agitation (200 rpm).

### Mutant Strain Construction

Allelic exchange mutagenesis was used to produce markerless deletions of the genes encoding two putative iron-sulfur proteins, *hucE* (MSMEG_2268) and *hhyE* (MSMEG_2718) (Supplementary Figure S2). Briefly, a fragment containing fused left and right flanks of the MSMEG_2268 (1800 bp) and MSMEG_2718 (3098 bp) genes were synthesized by GenScript. These fragments were cloned into the *Spe*I site of the mycobacterial shuttle plasmid pX33 (Gebhard et al., 2006) to yield the constructs pX33-*huc*E and pX33-*hhy*E (Supplementary Table S1). These constructs were propagated in *E. coli* TOP10 and transformed into wild-type *M. smegmatis* mc^2^155 cells by electroporation. Gentamycin (5 μg mL^–1^ for *M. smegmatis* or 20 μg mL^–1^ for *E. coli*) was used in selective solid and liquid medium to propagate pX33. Creation of the double iron-sulfur cluster mutant (Δ*hucE*Δ*hhyE*) was achieved by transformation of Δ*hhyE* electrocompetent *M. smegmatis* mc^2^155 with the pX33-*huc*E construct. Briefly, to allow for permissive temperature-sensitive vector replication, transformants were incubated on LBT gentamicin plates at 28°C until colonies were visible (5–7 days). Resultant catechol-positive colonies were subcultured onto fresh LBT gentamicin plates and incubated at 40°C for 3–5 days to facilitate integration of the recombinant plasmid flanks into the chromosome. The second recombination event was facilitated by subculturing catechol-reactive and gentamicin-resistant colonies onto LBT agar plates supplemented with 10% sucrose (w/v) and incubating at 40°C for 3–5 days. Catechol-unreactive colonies were subsequently screened by PCR to discern wild-type revertants from Δ*hucE*, Δ*hhyE* and Δ*hucE*Δ*hhyE* mutants. Primers used for the generation of mutants and for screening are listed in Supplementary Table S2.

### Complementation Vector Construction

The genes for the putative iron-sulfur proteins were amplified by PCR and the resulting fragments were cloned into the constitutive expression plasmid pMV261 *via Pst*I/*Hin*dIII site for *hucE* and *Bam*HI/*Hin*dIII site for *hhyE* (Stover et al., 1991) to yield the constructs pMV*hucE* and pMV*hhyE* (Supplementary Table S1). Sequence fidelity of the genes was verified through Sanger sequencing and insertion of the genes into the vector was confirmed through restriction-digestion analysis (Supplementary Figure S3). The plasmid constructs were propagated in *E. coli* DH5α and transformed into *M. smegmatis* cells by electroporation. Vector pMV*hucE* was transformed into *M. smegmatis* wild-type and Δ*hucE* strains, while pMV*hhyE* was transformed into wild type and Δ*hhyE* mutant. In addition, an empty pMV261 was transformed into wild-type, Δ*hucE*, and Δ*hhyE* strains. These seven *M. smegmatis* strains were used for complementation experiments in respirometry and activity staining. Kanamycin (20 μg mL^–1^ for *M. smegmatis* or 50 μg mL^–1^ for *E. coli*) was used in selective solid and liquid medium to propagate pMV261. Primers used for the generation of the constructs are listed in Supplementary Table S2.

### Respirometry Measurements

Cultures of wild-type, derived mutants, and complemented mutant strains of *M. smegmatis* were grown in 125 mL aerated conical flasks containing 30 mL HdB medium supplemented with 0.2% glycerol. Respirometry measurements were performed with mid-stationary phase cells, i.e., 72 h post OD_*max*_ (∼3.0). A Unisense H_2_ microsensor electrode was polarized at + 800 mV for 1 h using a Unisense multimeter and calibrated against standards of known H_2_ concentration. Gas-saturated PBS was prepared by bubbling the solution with 100% (v/v) of either H_2_ or O_2_ for 5 min. The 1.1 mL microrespiration assay chambers were sequentially amended with stationary-phase cultures (0.9 mL, OD_600_ = 3.0), H_2_-saturated PBS (0.1 mL), and O_2_-saturated PBS (0.1 mL). Chambers were stirred at 250 rpm, 37°C. Changes in H_2_ concentration were recorded using Unisense Logger Software, and upon observing a linear change in H_2_ concentration, rates of consumption were calculated over a period of 20 s, which corresponds to the most linear uptake of hydrogen by the cells. Oxidation rates were normalized against total protein concentration, which was determined by the bicinchoninic acid method (Smith et al., 1985) with bovine serum albumin standards.

### Activity Staining

Cultures of wild-type, derived mutants, and complemented mutant strains of *M. smegmatis* were grown in 2.5 L aerated conical flasks containing 500 mL HdB medium supplemented with 0.2% glycerol. For Huc activity staining, cultures of wild-type, Δ*hucS*, Δ*hucE*, Δ*hucS*Δ*hhyL*, Δ*hucE*Δ*hhyE*, and complemented Δ*hucE* and wild-type *M. smegmatis* (either with empty pMV261 or complementation vector pMV*hucE*) were harvested by centrifugation (10,000 × *g*, 10 min, 4°C) at early-stationary phase (24 h post OD_*max*_, ∼3.0) (Cordero et al., 2019b). For Hhy activity staining, cultures of wild-type, Δ*hhyL*, Δ*hhyE*, Δ*hucS*Δ*hhyL*, Δ*hucE*Δ*hhyE*, and complemented Δ*hhyE* and wild-type *M. smegmatis* (either with empty pMV261 or complementation vector pMV*hhyE*) were harvested by centrifugation at mid-stationary phase (72 h post OD_*max*_, ∼3.0) (Cordero et al., 2019b). Harvested cultures were washed in phosphate-buffered saline solution (PBS; 137 mM NaCl, 2.7 mM KCl, 10 mM Na_2_HPO_4_, 2 mM KH_2_PO_4_, pH 7.4), and resuspended in 16 mL lysis buffer (50 mM Tris-Cl, pH 8.0, 1 mM PMSF, 2 mM MgCl_2_, 5 mg mL^–1^ lysozyme, 40 μg mL^–1^ DNase, 10% glycerol). Resultant cell suspensions were passed through a Constant Systems cell disruptor (40,000 psi, four times), with unbroken cells removed by centrifugation (10,000 × *g*, 20 min, 4°C) to yield whole-cell lysates. Protein concentration was determined using a bicinchoninic acid assay with bovine serum albumin standards. Next, 20 μg of each whole-cell lysate was loaded onto two native 7.5% (w/v) Bis-Tris polyacrylamide gels prepared as described elsewhere (Walker, 2009) and run alongside a protein standard (NativeMark Unstained Protein Standard, Thermo Fisher Scientific) for 1.5 h at 25 mA. One gel was stained overnight at 4°C with gentle agitation using AcquaStain Protein Gel Stain (Bulldog Bio) for total protein determination. The other gel was incubated for hydrogenase activity staining in 50 mM potassium phosphate buffer (pH 7.0) supplemented with 500 μM nitroblue tetrazolium chloride (NBT) in an anaerobic jar amended with an anaerobic gas mixture (5% H_2_, 10% CO_2_, 85% N_2_ v/v) overnight at room temperature.

### Growth and Survival Assays

Cultures of wild type and derived mutants of *M. smegmatis* were inoculated into 125 mL conical flasks containing 30 mL LBT medium (initial OD_600_ of 0.001), in six biological replicates. Growth was monitored by measuring optical density at 600 nm (1 cm cuvettes; Eppendorf BioSpectrometer Basic); when OD_600_ was above 0.5, cultures were diluted ten-fold in LBT before measurement. Specific growth rate during mid-exponential growth was calculated for each replicate using GraphPad Prism (non-linear regression, exponential growth equation, least squares fit). The long-term survival of the cultures was determined by counting colony forming units (CFU mL^–1^) of cultures 21 days post-OD_*max*_. Cultures were serially diluted in HdB containing no carbon source and spotted on to agar plates in technical quadruplicates. After incubation at 37°C for 3 days, the resultant colonies were counted.

### Sequence and Phylogenetic Analysis

Sequences homologous to *M. smegmatis* HucE (MSMEG_2268) and HhyE (MSMEG_2718) were retrieved by protein BLAST (Altschul et al., 1990) using the National Center for Biotechnology Information (NCBI) Reference Sequence (RefSeq) database (Pruitt et al., 2007). The retrieved hits were cross-referenced with the hydrogenase database (HydDB) (Søndergaard et al., 2016) in order to determine which organisms co-encode HucE with HucL and HhyE with HhyL. For downstream phylogenetic and motif analysis, sequences were filtered to remove truncated HucE/HhyE proteins and retain one protein sequence per genus. This resulted in a representative subset of 52 full-length HucE and 26 full-length HhyE sequences. The retrieved sequences were aligned using ClustalW in MEGA7 (Kumar et al., 2016). The phylogenetic relationships of these sequences were visualized on a maximum-likelihood tree based on the Poisson correction method and bootstrapped with 100 replicates. In addition, WebLogo (Crooks et al., 2004) was used to analyze the conserved motifs containing cysteine and histidine residues predicted to bind iron-sulfur clusters. The web-based software Properon (M. Milton^1^) was used to generate to-scale genetic organization diagrams of the group 1h and group 2a [NiFe]-hydrogenases, with genes labeled according to the nomenclature in HydDB (Søndergaard et al., 2016).

## Results

### HucE and HhyE Are Predicted to Be Iron-Sulfur Proteins Associated With Group 2a and Group 1h [NiFe]-Hydrogenases

We investigated the diversity of putative iron-sulfur cluster proteins associated with [NiFe]-hydrogenases by conducting a homology-based search using the amino acid sequences of HucE (MSMEG_2268) and HhyE (MSMEG_2718) from *M. smegmatis* (Supplementary Figure S1). Homologous sequences were retrieved from 14 phyla and 104 genera of bacteria (Supplementary Figure S4 and Supplementary Table S3).

**FIGURE 1:**
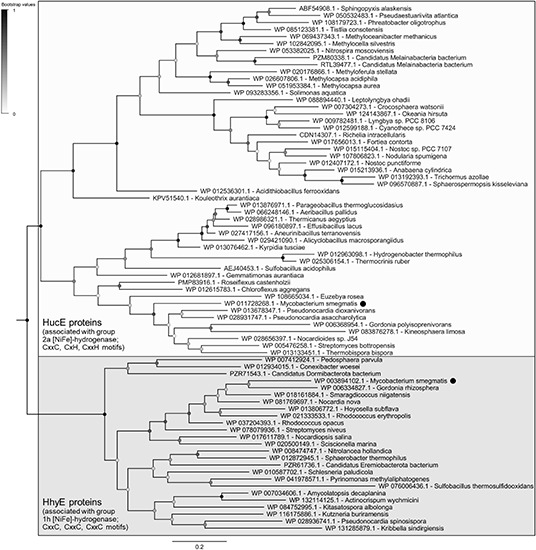
Phylogenetic tree of HucE and HhyE proteins associated with group 2a and 1h [NiFe]-hydrogenases. The tree visualizes the evolutionary relationships between a representative subset of 52 full-length HucE and 26 full-length HhyE sequences. The proteins encoded by *Mycobacterium smegmatis* are emphasized. The tree was constructed using the maximum-likelihood method (gaps treated with partial deletion), bootstrapped with 100 replicates, and rooted at the mid-point. The sequences used to create this tree are provided in Supplementary Table S3.

The evolutionary relationships of these proteins were visualized on a maximum-likelihood phylogenetic tree (Figure 1). All retrieved sequences fall into two robustly supported clades, the HucE proteins associated with group 2a [NiFe]-hydrogenases (Huc) and the HhyE proteins associated with group 1h [NiFe]-hydrogenases (Hhy), that share approximately 27% amino acid identity. HhyE proteins were encoded by various atmospheric H_2_ oxidizers, including *Streptomyces* (Berney and Cook, 2010), *Rhodococcus* (Meredith et al., 2014), *Pyrinomonas* (Greening et al., 2015a), and *Thermogemmatispora* (Islam et al., 2019). HucE proteins were encoded by various Cyanobacteria, which are known to recycle H_2_ produced during the nitrogenase reaction *via* group 2a [NiFe]-hydrogenases (Houchins and Burris, 1981; Tamagnini et al., 2002), as well as genera capable of aerobic hydrogenotrophic growth such as *Nitrospira* (Koch et al., 2014), *Pseudonocardia* (Grostern and Alvarez-Cohen, 2013), and *Acidithiobacillus* (Schröder et al., 2007). Of the hydrogenase-positive species surveyed, 9.5% lacked HucE and HhyE, including *Thermomicrobium* (Islam et al., 2019) and *Methylacidiphilum* (Mohammadi et al., 2017) species known to synthesize mid-affinity group 1h [NiFe]-hydrogenases. In contrast, no HucE or HhyE sequences were retrieved from organisms that lack hydrogenases.

Multiple sequence alignments show that HucE and HhyE proteins contain highly conserved motifs potentially involved in binding iron-sulfur clusters (Supplementary Figures S5, S6). Both HucE and HhyE contain a CxxC motif within a domain homologous to NifU proteins (Yuvaniyama et al., 2000). The C-terminus of HhyE proteins contains two CxxC motifs typical of iron-sulfur proteins (e.g., rubredoxins). In contrast, the HucE proteins contain an C-terminal motif CxH(x_15__–__18_)CxxC that matches the signature motif of Rieske iron-sulfur clusters (Schmidt and Shaw, 2001) (Supplementary Figure S6). A subset of the species surveyed contain truncated HucE and HhyE proteins that contain the NifU-like domain, but lack the C-terminal domains (Supplementary Figures S4, S5).

### HucE and HhyE Are Essential for H_2_ Oxidation in *Mycobacterium smegmatis*

We used allelic exchange mutagenesis to generate markerless single and double mutants of the *hucE* and *hhyE* genes in *M. smegmatis*, i.e., Δ*hucE*, Δ*hhyE*, and Δ*hucE*Δ*hhyE.* Gene deletion was confirmed by PCR targeting chromosomal sequences adjacent to the flanking regions used for homologous recombination (Supplementary Figure S2). Assays were used to compare H_2_ oxidation of these strains with the wild-type strain and strains containing previously generated deletions of the hydrogenase structural subunits, i.e., Δ*hucS*, Δ*hhyL*, and Δ*hucS*Δ*hhyL*, that lack hydrogenase activity (Berney and Cook, 2010; Berney et al., 2014b; Greening et al., 2014a).

**FIGURE 2:**
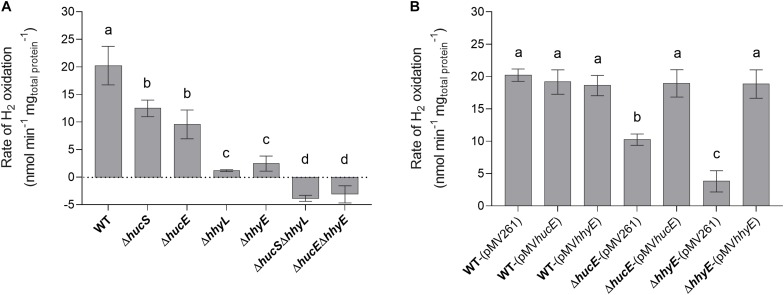
Hydrogen oxidation by wild-type, derived mutants, and complemented mutant strains of *M. smegmatis*. H_2_ uptake by whole cells in mid-stationary phase (72 h post OD_*max*_ ∼3.0) was measured amperometrically using a Unisense H_2_ electrode. **(A)** Comparison of the rates of H_2_ oxidation between wild-type, single and double mutants of the iron-sulfur proteins (Δ*hucE*, Δ*hhyE*, Δ*hucE*Δ*hhyE*), and single and double mutants of hydrogenase structural subunits (Δ*hucS*, Δ*hhyL*, Δ*hucS*Δ*hhyL*). **(B)** Rates of H_2_ oxidation in Δ*hucE* and Δ*hhyE* strains complemented with expression of *hucE* and *hhyE*, respectively. Controls include wild-type, Δ*hucE*, and Δ*hhyE* strains transformed with empty vector pMV261 and wild-type strain transformed with complementation vectors pMV*hucE* and pMV*hhyE*. Error bars show standard deviations of three biological replicates and values labeled with different letters are significantly different (*p* < 0.05) based on a one-way ANOVA.

We first used a H_2_ electrode to measure rates of aerobic H_2_ respiration mediated by whole cells of each strain. There were significant differences in the rate of H_2_ oxidation for all deletion strains compared to the wild type (Figure 2A). Loss of *hucE* and *hhyE* resulted in reductions of 1.8-fold and 8.4-fold, respectively; such reductions were statistically indistinguishable from those observed in the mutants of the hydrogenase structural subunits *hucS* and *hhyL*. Deletion of both iron-sulfur proteins (Δ*hucE*Δ*hhyE*) or both hydrogenase structural subunits (Δ*hucS*Δ*hhyL*) caused complete cessation of H_2_ oxidation, highlighting that these two hydrogenases are solely responsible for H_2_ oxidation and that the putative iron-sulfur proteins are indispensable for this process. The low-level negative rates in Δ*hucE*Δ*hhyE* and Δ*hucS*Δ*hhyL* strains most likely reflect drift of the electrode rather than actual H_2_ production by Hyh (Hyd3), since this hydrogenase is only upregulated during hypoxia (Berney et al., 2014a). We successfully complemented the Δ*hucE* and Δ*hhyE* strains by reintroducing the *hucE* and *hhyE* genes on the episomal plasmid pMV261 (Stover et al., 1991) (Figure 2B); in contrast, introducing the empty vector caused no effect and neither did introducing the complementation vectors in a wild-type background. This restoration of Huc and Hhy activities in complemented iron-sulfur protein deletion mutants strongly indicate that HucE and HhyE are essential for H_2_ oxidation. Moreover, the similarity in H_2_ oxidation rates between the strains containing deletions of the catalytic subunits, compared to the putative iron-sulfur proteins, is consistent with HucE and HhyE being functionally linked with the Huc and Hhy hydrogenases, respectively.

**FIGURE 3:**
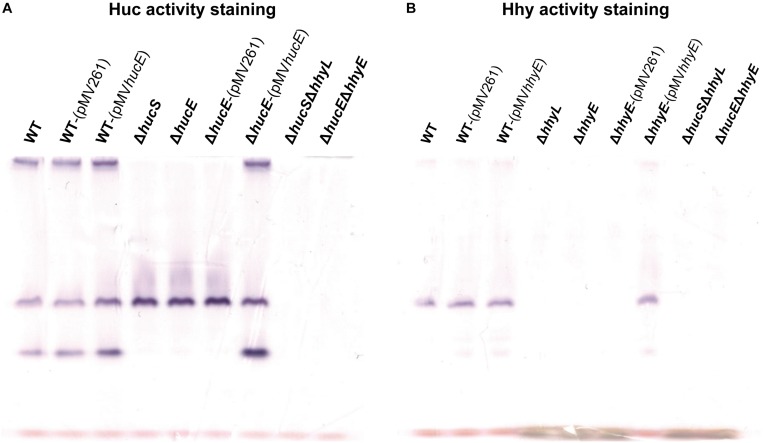
Hydrogenase activity staining in wild-type, derived mutants, and complemented mutant strains of *M. smegmatis.* Whole-cell lysates were used for zymographic staining of H_2_ uptake in a H_2_-rich atmosphere with nitroblue tetrazolium as artificial electron acceptor. **(A)** Huc activity staining of cultures of wild-type, Δ*hucS*, Δ*hucE*, Δ*hucS*Δ*hhyL*, Δ*hucE*Δ*hhyE*, and complemented Δ*hucE* and wild-type *M. smegmatis* (either with empty pMV261 or complementation vector pMV*hucE*) harvested at early-stationary phase (24 h post OD_*max*_ ∼3.0). **(B)** Hhy activity staining of cultures of wild-type, Δ*hhyL*, Δ*hhyE*, Δ*hucS*Δ*hhyL*, Δ*hucE*Δ*hhyE*, and complemented Δ*hhyE* and wild-type *M. smegmatis* (either with empty pMV261 or complementation vector pMV*hhyE*) harvested at mid-stationary phase (72 h post OD_*max*_ ∼3.0). The original gels and Coomassie stain are shown in Supplementary Figure S7.

In an interrelated assay, we performed activity staining of the Huc and Hhy hydrogenases using whole-cell lysates of wild-type and deletion mutant strains, with and without the complementation vectors, in the presence of the artificial electron acceptor nitroblue tetrazolium chloride. In the Huc activity staining gel (Figure 3A), three bands were observed in the whole-cell lysates of wild-type strains, with or without complementation vectors: the top high-MW band, middle mid-MW band, and bottom low-MW band. Both the high-MW and low-MW bands correspond to Huc activity (Cordero et al., 2019b) and these bands were not observed in strains lacking either *hucS* or *hucE*. However, Huc activity was restored when the Δ*hucE* strain was complemented by episomal expression of *hucE*. For Hhy activity staining (Figure 3B), a mid-sized MW band was be observed in all wild-type strains. This band, which is the same middle band observed in the Huc activity stain (Figure 3A), corresponds to Hhy activity (Greening et al., 2014a; Cordero et al., 2019b). No Hhy staining was detected with the loss of either *hhyL* or *hhyE*, but complementation of the Δ*hhyE* strain with *hhyE* restored Hhy activity. The similarity in the staining bands observed between Δ*hucS* and Δ*hucE* strains or between Δ*hhyL* and Δ*hhyE* indicate that the putative iron-sulfur proteins HucE and HhyE, like their respective hydrogenase core subunits HucS and HhyL, are important for hydrogenase activity. The artificial electron acceptor cannot compensate for the loss of HucE/HhyE and neither can HucE for HhyE nor HhyE for HucE. This further supports the model that HucE and HhyE form a functional association with Huc and Hhy, respectively.

### HucE and HhyE Mutant Strains Have Significant Growth and Survival Defects

**FIGURE 4:**
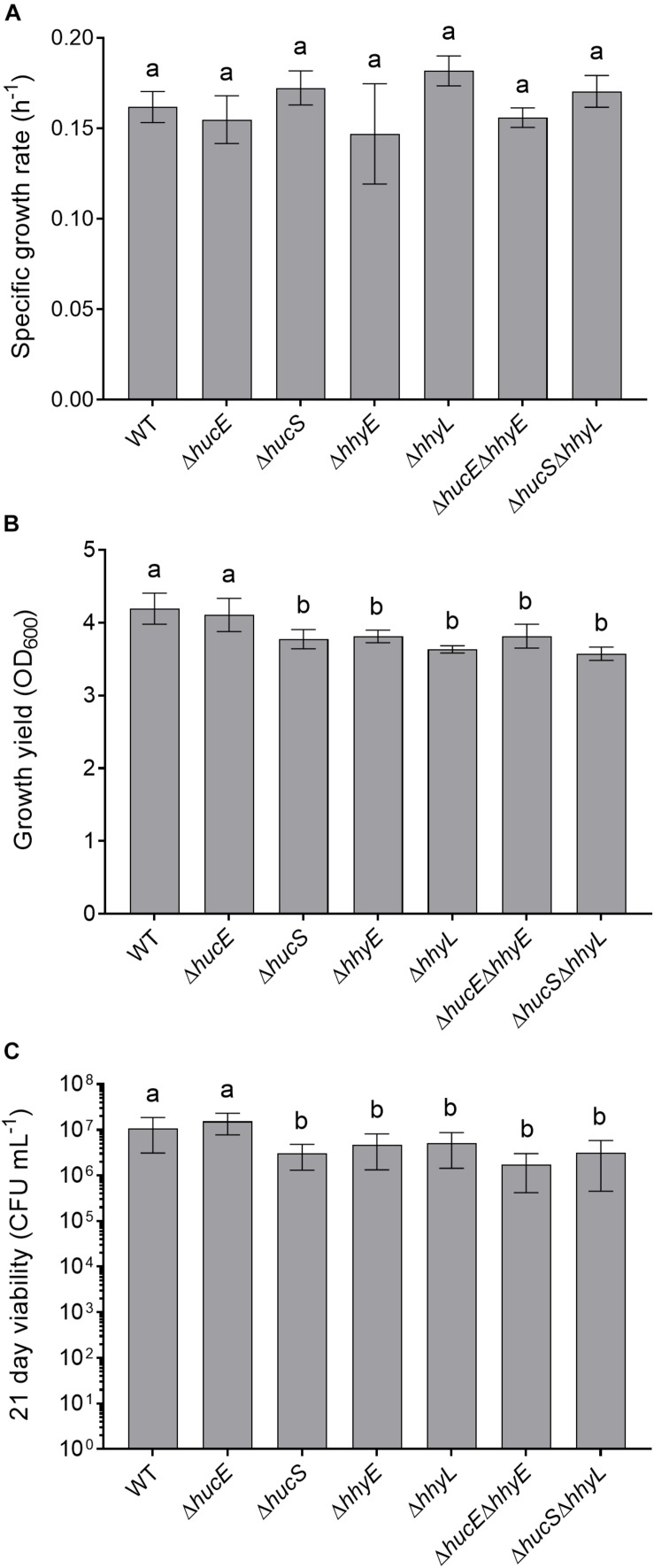
Comparison of growth and survival between wild-type and mutant strains of *M. smegmatis*. Seven strains were grown on lysogeny broth supplemented with Tween80 (LBT): wild-type, single and double mutants of the iron-sulfur proteins (Δ*hucE*, Δ*hhyE*, Δ*hucE*Δ*hhyE*), and single and double mutants of hydrogenase structural subunits (Δ*hucS*, Δ*hhyL*, Δ*hucS*Δ*hhyL*). **(A)** Specific growth rate (μ) during exponential phase. **(B)** Final growth yield (OD_*max*_) at 24 h post-stationary phase. **(C)** Long-term survival (CFU mL^– 1^) at 21 days post-stationary phase. Error bars show standard deviations of six biological replicates. Values labeled with different letters are significantly different (*p* < 0.05) based on a one-way ANOVA.

Previous genetic studies have shown that the hydrogenases modestly increase growth yield and long-term survival of *M. smegmatis* (Berney and Cook, 2010; Greening et al., 2014b). We therefore tested whether these findings extended to the putative iron-sulfur proteins by analyzing the growth rate, growth yield, and long-term survival of the seven aforementioned strains when cultured aerobically on rich media (LBT). In line with previous findings (Berney et al., 2014a; Greening et al., 2014b), no significant differences in specific growth rate were observed between the strains (Figure 4A). However, there was a 10% reduction in the specific growth yield of the HhyE mutant compared to the wild-type strain (OD_*max*wt_ = 4.19 ± 0.21; OD_*max*_ Δ*hhyE* = 3.81 ± 0.09; *p* = 0.008) (Figure 4B). This phenotype extended to the double mutant strain (Δ*hucE*Δ*hhyE*) and again phenocopied single and double mutants lacking the *hhyL* gene.

We also tested whether the strains were defective in long-term survival by counting colonies of aerobic cultures 21 days following OD_*max*_. There were significant reductions in the survival of most strains compared to the wild-type (Figure 4C). Cell counts were approximately two-fold lower for the Δ*hhyE* and Δ*hhyL* strains (*p* < 0.02), and four-fold lower for the double mutant strains (*p* < 0.002), relative to the wild-type. These findings agree with previous reports that atmospheric H_2_ oxidation by the hydrogenases enables *M. smegmatis* to survive energy starvation (Greening et al., 2014b) and further supports that the putative iron-sulfur proteins contribute to this function. For reasons currently unclear, no phenotypes were observed for the Δ*hucE* strain.

## Discussion

In summary, this study shows that HucE and HhyE are required for the enzymatic activity and physiological function of the mycobacterial uptake hydrogenases. Strains lacking these proteins showed no hydrogenase activity in either amperometric or zymographic assays. Furthermore, they exhibited growth and survival phenotypes similar to those of knockouts of hydrogenase structural subunits (Berney and Cook, 2010; Greening et al., 2014b); as with the structural subunit mutants, these phenotypes are relatively minor, likely reflecting the numerous survival mechanisms present in *M. smegmatis* such as the ability to persist on carbon monoxide (Cordero et al., 2019a). Despite some sequence similarity between the two proteins, they are non-redundant, as there was no compensation in hydrogenase activity in the single mutant strains. The genomic survey and phylogenetic analysis indicate that *hucE* and *hhyE* genes co-evolved with the genes encoding the structural subunits of the group 2a and group 1h [NiFe]-hydrogenases. Their detection in the genomes of most but not all characterized high-affinity H_2_ oxidizers indicate they are important but overlooked mediators of atmospheric H_2_ oxidation. They are also associated with the group 2a [NiFe]-hydrogenases of H_2_-recycling Cyanobacteria and various aerobic hydrogenotrophic bacteria that are not currently known to oxidize atmospheric H_2_.

This study lends some support to the hypothesis that these proteins serve as the immediate electron acceptors for the group 2a and group 1h [NiFe]-hydrogenases. There are broadly five lines of evidence that support this hypothesis: (i) the presence of highly conserved motifs for binding iron-sulfur clusters, (ii) the essentiality of these proteins for the function of these hydrogenases, (iii) their association with the structural rather than maturation operons of the hydrogenases (Berney et al., 2014b), (iv) co-localization of HhyL, HhyS, and HhyE subunits on native polyacrylamide gels (Cordero et al., 2019b), and (v) their genomic association with hydrogenases that lack known electron transfer subunits (e.g., cytochrome *b* subunits). With the respect to the latter point, it is interesting that these proteins are conserved in Cyanobacteria, given the immediate electron acceptors of their uptake hydrogenases have long remained enigmatic (Tamagnini et al., 2002). It is also notable that HucE proteins encode the signature motifs of a Rieske iron-sulfur cluster. Given their unusual ligands, these clusters have a higher standard redox potential (*E*_*o*_’ > −150 mV) than most iron-sulfur clusters (e.g., ferredoxins) (Brown et al., 2008). They would therefore be well-poised to accept the relatively high-potential electrons derived from atmospheric H_2_ and transfer them to menaquinone. Consistently, zymographic studies suggest that the high-affinity hydrogenases operate at higher redox potential than prototypical hydrogenases, given they are reactive with the nitroblue tetrazolium (*E*_*o*_’ = −80 mV) but not viologen compounds (*E*_*o*_’ = −360 mV) (Pinske et al., 2012; Greening et al., 2014a).

While this study demonstrates HucE and HhyE are important for mycobacterial hydrogenase activity, further work is ultimately needed to resolve their respective function. While a role in electron transfer is most plausible, we have not demonstrated that these proteins interact with the hydrogenases and it is notable that the artificial electron acceptor nitroblue tetrazolium chloride cannot compensate for their absence. In this regard, other roles are also possible and compatible with the available evidence, for example as specific assembly factors and/or structural scaffolds for the hydrogenases. For example, it has been demonstrated that a rubredoxin-related protein is important for aerobic maturation of the group 1d [NiFe]-hydrogenase in *R. eutropha* (Fritsch et al., 2014). Furthermore, it is possible that other hypothetical proteins downstream of HucE and HhyE may also serve as electron acceptor candidates, in particular MSMEG_2717 that shares homology to PHG067, the proposed electron acceptor of *R. eutropha* (Schäfer et al., 2013). Biochemical studies, including studying the redox chemistry of these proteins and their interactions with the as-yet-unpurified hydrogenases, are now required to distinguish these possibilities and develop a sophisticated understanding of their function.

## Data Availability Statement

All datasets generated for this study are included in the article/Supplementary Material.

## Author Contributions

CG conceived, designed, and supervised the study. CG and ZI were responsible for the phylogenetic analysis and analyzed the data. ZI and PC were responsible for the knockout generation, hydrogen electrode measurements, and activity staining. PC was responsible for the complementation experiments. ZI was responsible for the phenotypic assays. ZI, PC, and CG wrote the manuscript.

## Conflict of Interest

The authors declare that the research was conducted in the absence of any commercial or financial relationships that could be construed as a potential conflict of interest.

## Footnotes

1 https://zenodo.org/record/3519494#.Xb4CnJozZPY
