# Supplementary material for: Putative Iron-Sulfur Proteins Are Required for Hydrogen Consumption and Enhance Survival of Mycobacteria
Source: Front Microbiol. 2019 Nov 22;10:2749. doi: 10.3389/fmicb.2019.02749 (PMC6883350; doi:10.3389/fmicb.2019.02749)
Supplement: Supplementary file 1 [file Data_Sheet_1.PDF]

Supporting Information

Figure S1. Genes associated with Huc and Hhy in *Mycobacterium smegmatis*. (**a**) Organisation of the genes associated with the group 1h [NiFe]-hydrogenase, Hhy. (**b**) Organisation of the genes associated with the group 2a [NiFe]-hydrogenase, Huc. Abbreviations: HMP = hydrogenase maturation protease; NHL = NHL repeat protein; GmhA = phosphoheptose isomerase homolog; AraC = arabinose regulatory protein homolog. The length of the genes is shown to scale. The red line is used to denote genes that are transcribed in operons, as verified by previous RT-PCR studies (Berney et al., 2014b).

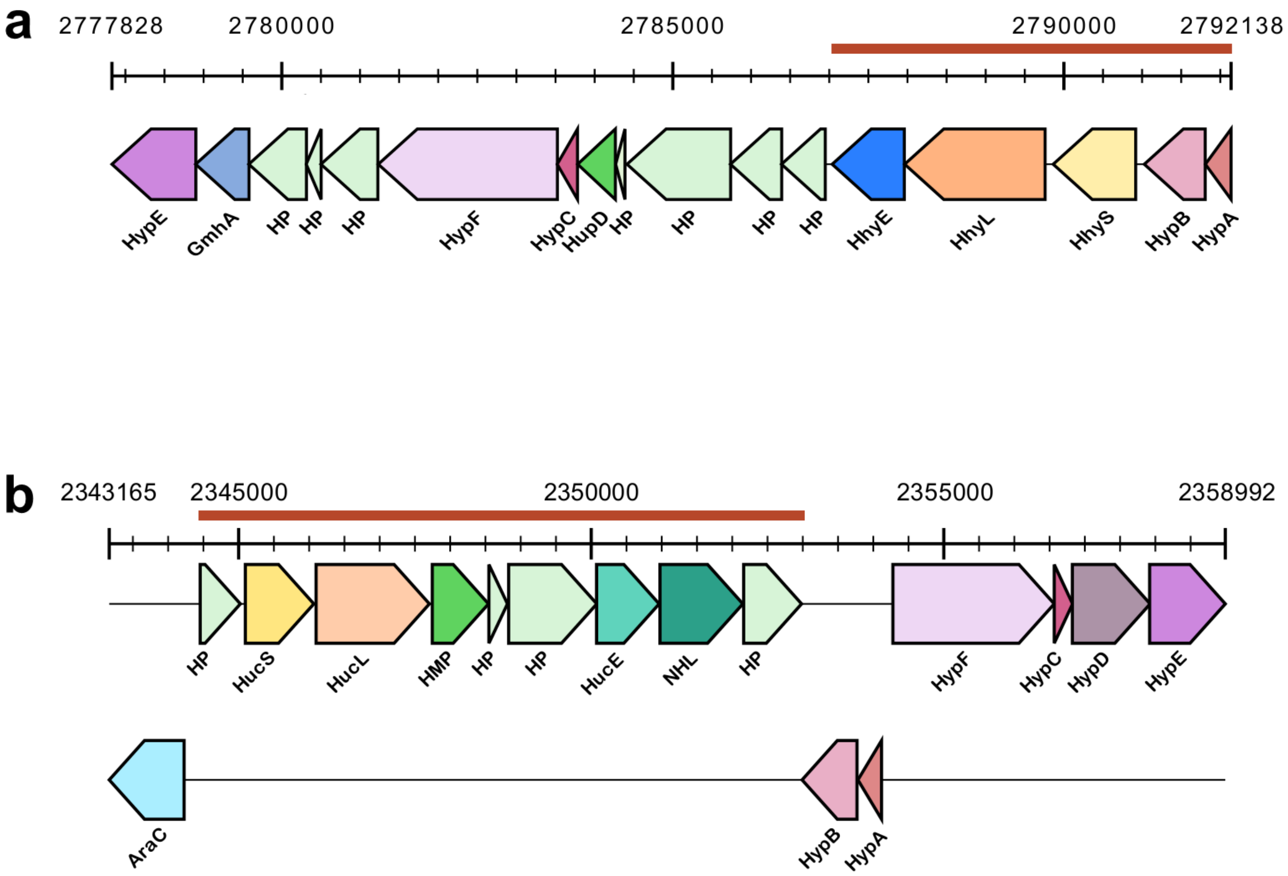

Figure S2. Deletion of the *hucE* and *hhyE* genes in *Mycobacterium smegmatis*. The schematic shows the four main steps that led to the production of knockouts of (**a**) *hucE* (MSMEG_2268) and (**b**) *hhyE* (MSMEG_2718). (**i**) Construction of the pX33-*hucE* / pX33-*hhyE* vectors containing a fused left flank (LF) and right flank (RF) of each gene. (**ii**) Temperature-mediated integration of the vector into the *M. smegmatis* chromosome *via* either the left flank or right flank of the chromosomal *hucE* / *hhyE* genes. (**iii**) Chromosomal excision of the vector due to *sacB*-mediated sucrose toxicity to either wild-type revertants or

Δ*hucE* / Δ*hhyE* mutants. (**iv**) PCR-based screening through primers (2267_chrom_fwd, 2269_chrom_rvs, 2717_chrom_fwd, and 2719_chrom_rvs) targeting the flanks to confirm whether colonies are (**a**) wild-type revertant (2774 bp product) or Δ*hucE* mutant (1890 bp product) and (**b**) wild-type revertant (4110 bp product) or Δ*hhyE* mutant (3183 bp product). Both primer sets were used to confirm the double mutant.

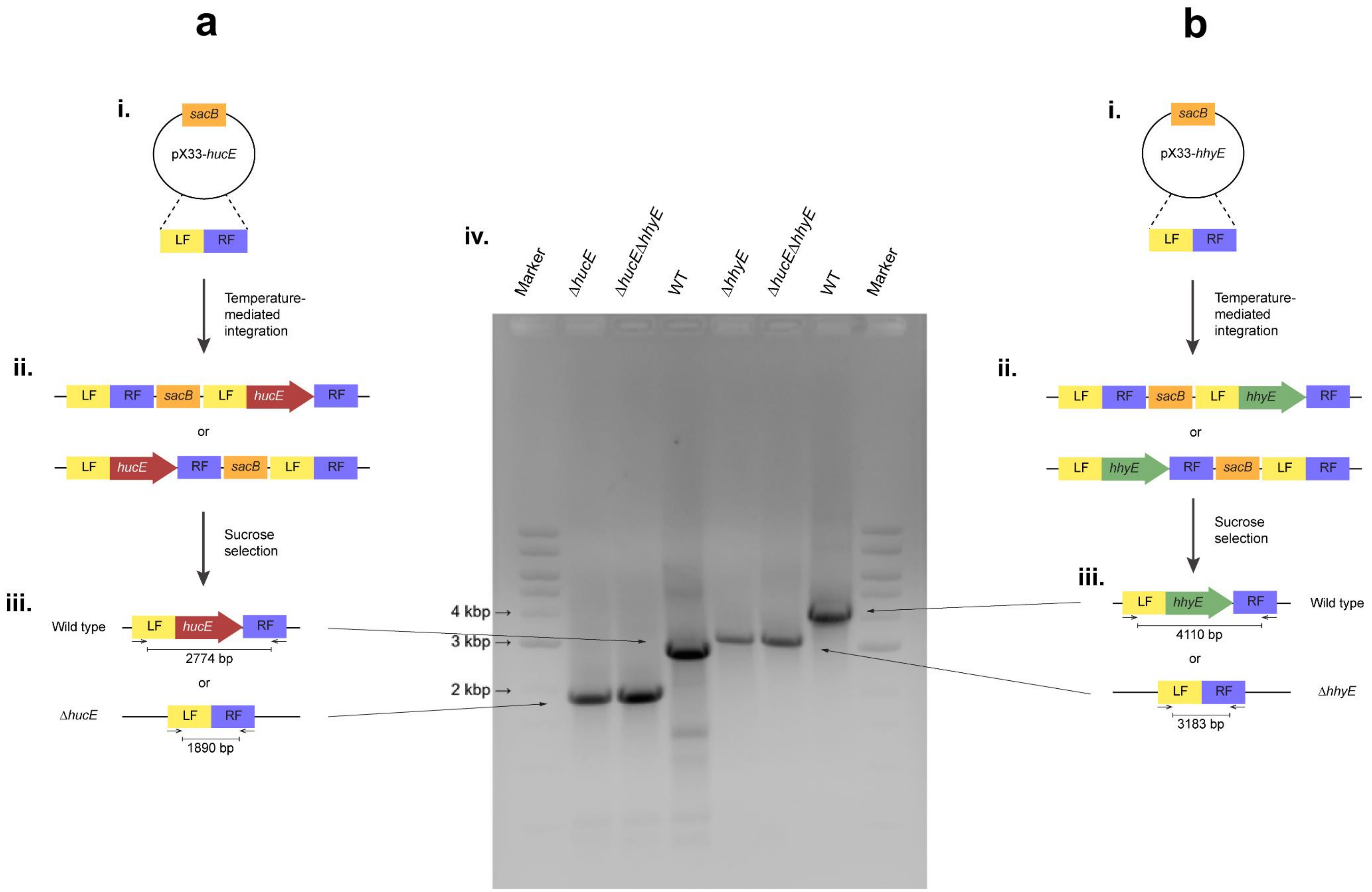

a
i.
sacB
pX33-hucE
LF RF
Temperature-mediated integration
ii.
LF RF sacB LF hucE RF
or
LF hucE RF sacB LF RF
Sucrose selection
iii.
Wild type
LF hucE RF
2774 bp
or
ΔhucE
LF RF
1890 bp
iv.
Marker
ΔhucE
ΔhucEΔhhyE
WT
ΔhhyE
ΔhucEΔhhyE
WT
Marker
4 kbp
3 kbp
2 kbp
b
i.
sacB
pX33-hhyE
LF RF
Temperature-mediated integration
ii.
LF RF sacB LF hhyE RF
or
LF hhyE RF sacB LF RF
Sucrose selection
iii.
LF hhyE RF
Wild type
4110 bp
or
LF RF
ΔhhyE
3183 bp

Figure S3. Construction of complementation vectors. (**a**) pMV*hucE* digested with PstI and HindIII (left side) produced two fragments (893 bp and 4480 bp) consistent with *hucE* insertion to the plasmid. Map of the plasmid is shown on the right side. (**b**) pMV*hhyE* digested with BamHI and HindIII produced two fragments (932 bp and 4468 bp) consistent with *hhyE* insertion to the plasmid. Map of the plasmid is shown on the right side. The plasmids have an *Escherichia coli* pBR322 origin of replication, a mycobacterial OriM derived from pAL5000, a kanamycin resistance cassette (KanR), and a promoter P_{hsp60}.

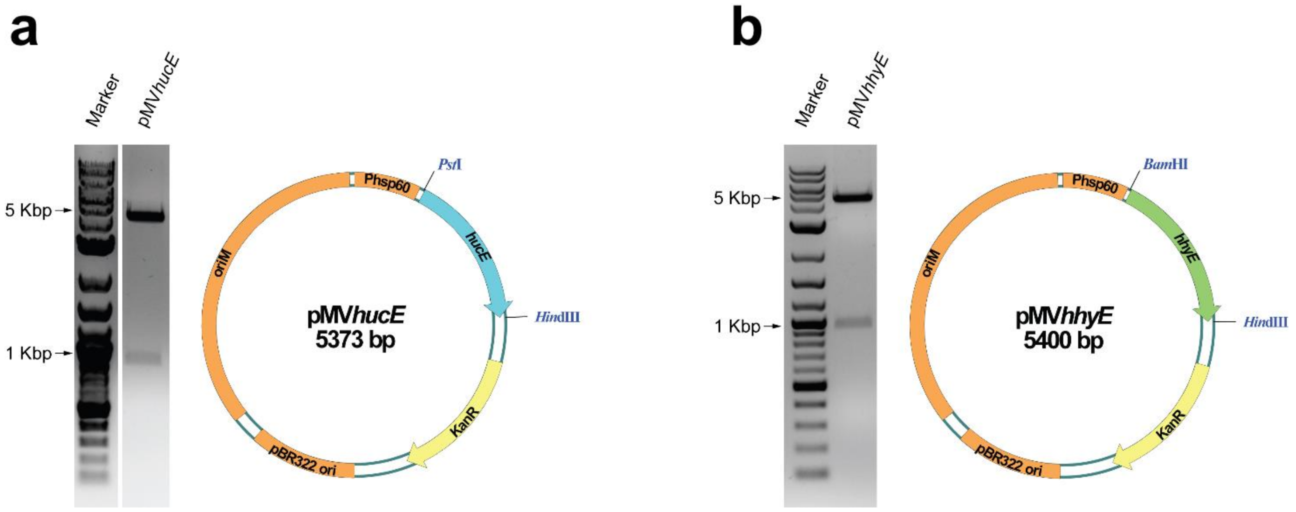

Figure S4. Distribution of HucE and HhyE proteins in hydrogenase-encoding microorganisms. (**a**) Distribution of HucE proteins in genomes of bacteria that encode group 2a [NiFe]-hydrogenases. (**b**) Distribution of HhyE proteins in genomes of bacteria and archaea that encode group 1h [NiFe]-hydrogenases. Genomes are coloured depending on whether a full-length protein is present, a truncated protein is present, or the protein is absent.

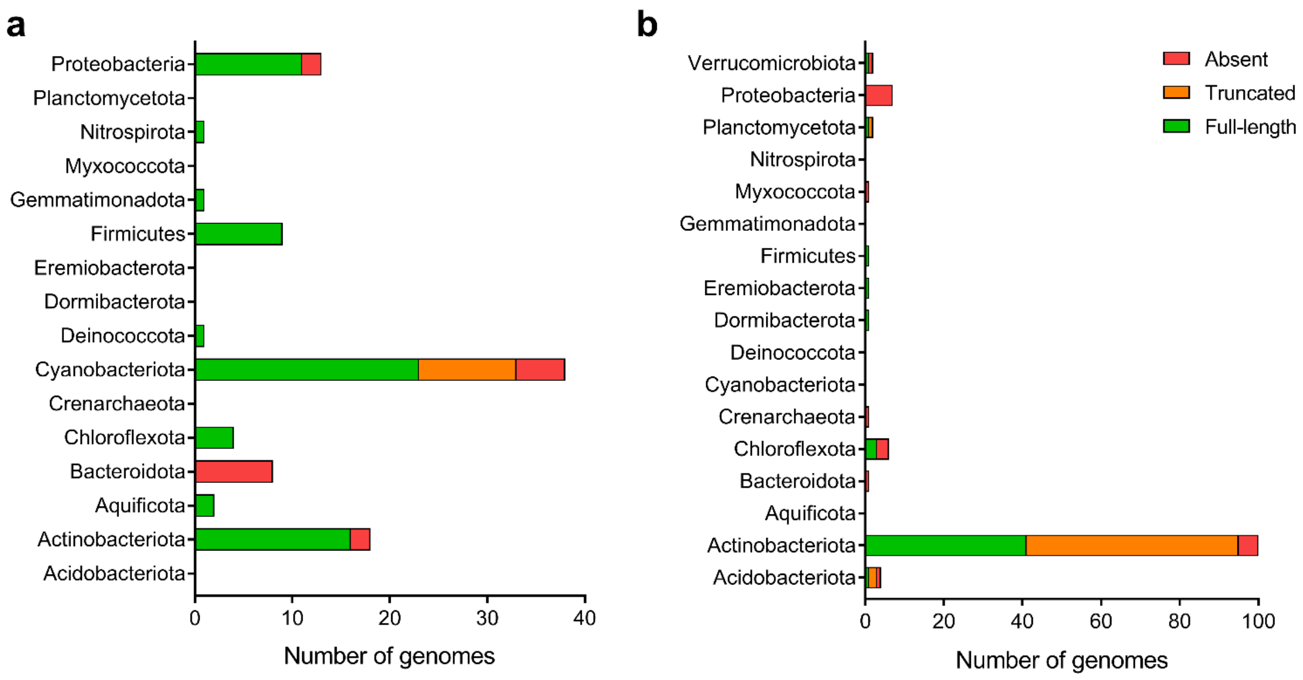

Figure S5. Multiple sequence alignment of the HucE and HhyE family proteins from bacteria that have been shown or predicted to mediate aerobic H_2 respiration. The proteins shown are: WP_011728268.1 (*Mycobacterium smegmatis* HucE), WP_013678347.1 (*Pseudonocardia dioxanivorans* HucE), WP_053382025.1 (*Nitrospira moscoviensis* HucE), WP_012963098.1 (*Hydrogenobacter thermophilus* HucE), WP_015213936.1 (*Anabaena cylindrica* HucE), WP_015115404.1 (*Nostoc* sp. PCC 7107 HucE), WP_016860574.1 (*Fischerella muscicola* HucE truncated), WP_003894102.1 (*Mycobacterium smegmatis* HhyE), WP_041978571.1 (*Pyrinomonas methylaliphatogenes* HhyE), WP_084922766.1 (*Rhodococcus equi* HhyE), WP_010988763.1 (*Streptomyces avermitilis* HhyE truncated), PZR71543.1 (*Candidatus* Dormibacterota bacterium HhyE), PZR61736.1 (*Candidatus* Eremiobacterota bacterium HhyE). The conserved cysteine and histidine residues predicted to bind iron-sulfur clusters are coloured in red and the motifs are highlighted.

```
WP_011728268.1      ----------------------MTAAVEAPELEKLAQRVDNAVRA--LVDLDPEARKVAE     36
WP_013678347.1      --------MTASTKSTPAASSGPAATEAEPSFEDLAGRVDRAVEA--ASALDGAAGEAAA     50
WP_053382025.1      -----------------MEKQESTVLETQPTLASLVKSIKSLEVI--VEGWNDSQRLTVQ     41
WP_012963098.1      -------------------------MTEELSFEKLAERIDELLAK--VKNFEDEKRETVG     33
WP_015213936.1      ----------------------------MNQLEELVEDINRFEAI--IAEWDESQRCVAV     30
WP_015115404.1      ----------------------------MTNLEEFVEEINRFEAI--ISEWDESQRCVAV     30
WP_016860574.1      ----------------------------MTNLEELVQEISRFEAI--ISEWEESQRCVAI     30
WP_003894102.1      ---MAPAVTPLADVP--VDT---Q-SDDEARWRTAGERIESLLDA----AAGPTARARAE     47
WP_041978571.1      -----------------MEQ---QMQACARRIEELVQRIESLTD--------ARARDVAV     32
WP_084922766.1      --------MASAHDE--TRY---E-TPESDRWRQAGDRIEALLDA--TSAGGPVARERAE     44
WP_010988763.1      --------MSAVS------------ATNRVTAEQTGRRIEDVLDRLAA-TGDPAAGAAAE     39
PZR71543.1          --------MAIL-----QRK---EFDRRLQQTESLINEVEALAD--------EEVKRRAV     36
PZR61736.1          MDERTPALTEGHDRRSAPPD---HLARRPEDPRALGDRVDALLQQLATSPNPAHARAKAE     57
```

```
                                                          :.                  .

WP_011728268.1      ELKAALEEIHRVGLVTIVKRMRSGE--------ATREALFELVDDPVVRMLFTLHGIIRP     88
WP_013678347.1      ELRAAIEEIHRVGLRAIVRAMRERP--------ETRDLLFTLVDDPVVHLLLSLHGIVRP     102
WP_053382025.1      ALRKAIEDLHKEAFSRLIRGIKADP--------AAIPALRQAVGDEIVYAVLRHLGLLKP     93
WP_012963098.1      ELIKSIEEFTRMALVKLVKLMKEDS--------AGKDILLKAVREPEIYSLFLKHGIIRE     85
WP_015213936.1      GLKRSIEALHKAALTNLIKSLKQN----------NMSGLRDAVSDEIVYAVLLYHELVKP     80
WP_015115404.1      GLKRAIEALHKEALTRLIKSLKQE----------SISALRQAADDEVVYAVLLYHDLVKP     80
WP_016860574.1      GLKRAIEDLHKEALTRLIKSVKQE----------SISALRNAVQDEVVYGVLLYHELVKS     80
WP_003894102.1      QLVREVTDLYGAALERMLRAAV----------TAAPELAETFAADDLVASLLLVHGLHPH     97
WP_041978571.1      ELVQELMRMHGAALERLLEIVWG---------RNEPELIEAFARDDLVGPLLLLYDLHPL     83
WP_084922766.1      QLVREVVDLYGEALGRVLTIAA-----------RAPGLVDELARDELVSSMLLVGGLHPH     93
WP_010988763.1      ELVRTLMEFYGSGLARILHLLSSAPGG----PRPPGDPLAALLGDELVASLLVLHDLHPE     95
PZR71543.1          EAIQALLNLHGEGLERTLELISDS-S------NQGQALIDELGKDPVVGGLLLLHGVHPL     89
PZR61736.1          ELVSTLVRLYGAGLTRILEIVDENAG------DSGGRIFRHLCDDDLVASLLILHGLHPL     111
                         :  :   .:   :                          :  :  ::     :

WP_011728268.1      DT---TTLAEQALAQVRPQLRSHGGDVTLVRI---ES-GTAFVRL-EGACNGCSMSAVTL     140
WP_013678347.1      DP---ATHAQRVLTTVRPQLQSHGGDVAFAHV---AD-GVAYVRL-EGACNGCSMSAVTL     154
WP_053382025.1      S---LNERIERALASVRPLLQGHGGNVELVRV---DPPDSVEIKL-LGACDGCPASGLTL     146
WP_012963098.1      DD---RTKAIKALELIRPYIRSHGGDVEFVDL---KE-KTLYVRL-RGTCTGCSQVSFTL     137
WP_015213936.1      P---LAERIQTALAEVRPGLQSHNGDIELVAI---KPPDTVEVKL-IGSCSSCPSSTLTL     133
WP_015115404.1      PLPPILQRIQAALEEVRPGLKSHNGDIELVAF---KPPDTVEVKL-IGTCSSCPASHLTL     136
WP_016860574.1      PTPPLQQRIQQALEEVRPGLKSHNGDVELVAI---KAPDTVEVKL-IGTCGNCPASTLTL     136
WP_003894102.1      P---VHRRIADALDRVRPYLGSHGGDVRLLDVVPEADGAVARLRF-SGSCKSCPSSAATL     153
WP_041978571.1      D---QETRVREAIERVRPYLRSHGGDVELLRI----SDGVVHLRL-RGSCHGCASSAQTL     135
WP_084922766.1      D---VETRVRTALDSVRPYLGSHGGDVELVDVT---DDGVVRLRL-LGSCHGCPSSAVTL     146
WP_010988763.1      D---RDVRIARALDSVRDQAL----EVVAFD---ETSGTLRLRTRAGAGC-GCAGTGTAA     144
PZR71543.1          G---LEDRVLQALDKVRPYLGSHGGNVQLVGL----DEGVVRLRL-EGTCQGCASSAVTL     141
PZR61736.1          S---LEERIVQALDRVRVYLKSHEGDVQLLRI----EGETVYLRM-AGSCHGCPSSASTM     163
                               .:  :*        ::               ::   *:  .*

WP_011728268.1      RQLVETALLEGVQGVSKVEVLPNE-----PTPTVISIDAVLAGSTVRRRAEEGWVRAGSV     195
WP_013678347.1      RNLVEEALVAQVPAIRSVEVLPNE-----PTPTLISVESMFPRST---GLPPGWVDGLGT     206
WP_053382025.1      AEGIEKAIREHCPEIITIKKVKGI-----PPSSNVNGASVFFVSPFARSSDAGWQFAVEL     201
WP_012963098.1      QQTILEAVQAYIPHIEKVELAKDT-----PVEAFLELS----------GKEEGYIKAFDI     182
WP_015213936.1      TQGVEEAIKKHCPEITKVIAVN--------SSPNVT--ETNFNSPFPLPTDFYWVRVATV     183
WP_015115404.1      SQGVEQSIKNYCPEINNVVAVG--------DRSTVDNTNTGLTTPFPPTITTAWVRVATV     188
WP_016860574.1      SQGIEQTIKTHCPEINHVIAVS--------------------------------------     158
WP_003894102.1      ELAISDAVLAAAPEVSSIEVVNAEPEP----AAVIPAQSLLARVHSDGRRHVEWHAVPEL     209
WP_041978571.1      RLAIEEAAIYEAAPEIAALEVEGVAQ----PATSFVPLESGRGNANGKRKTHGVWEEVEAP     191
WP_084922766.1      QLAVEGAVQAAAPETTAIEVETEDRETA-PTPGVFTVDSLMSHVREEPTRTGTWLAAEEF     205
WP_010988763.1      VQAAEDALACFAPEVTAVEMQPAAAPG-EPALLQIGTGPNSGAVARPAAAKTA-------     196
PZR71543.1          KFAVEREIMEAAPDVVAIEVADDSAER--PPEGFIPLGQIKPLA--PKPASTDWEVVAGV     197
PZR61736.1          KLAIERAIHDAAPEITEIRAEGVHDGA-------------------VTRKPTEWVSLAAL     204
                           :         :

WP_011728268.1      DAAAVDDVTPVTVTAPDGSEVRLLLVNLGQRMSAYRNECAHEALPLDGAVL------DVG     249
WP_013678347.1      DDVAPGALAAVQLAADDGTGADVIVVNVDNRISAYRNACAHEGLPLDGALL------DVR     260
WP_053382025.1      AEIPDIGIK-----VLELAGHSLLFSRTDQVVTCFENACAHMGMPLDMGLI--------A     248
WP_012963098.1      SELKEGHVY-----RFLHEGVDVILMLWKGRVYAYRNSCAHQGHPLHEGEL------T-E     230
WP_015213936.1      DQINKSSVL-----SVRIANQDLILNRQGENITCYRNSCSHLGYPLDKGKV--------E     230
WP_015115404.1      EQIPDLGIY-----APKVAGHALILYRQGDRITCYRNACSHLASPLDTGKV--------E     235
WP_016860574.1      ------------------------------------------------------------     158
WP_003894102.1      DGLSPGQVG-----GFRVDGATVMACRVGDDYFAYHDRCAWCGSTFAGAVL--------T     256
WP_041978571.1      LSLAPGSAR-----IIEARGRRILFCRLDDRFFAYEDRCPACGGALDAARF--------E     238
WP_084922766.1      AELAPGEVG-----GFTVGGLSMLVCRIGDELFAYRDRCPACGNSMAGTSLHRRAGGHVN     260
WP_010988763.1      ------------------------------------------------------------     196
PZR71543.1          GHLESGSTK-----VVRVGGSAVLFCSWGETTFAYRDSCPACGASLVGGDL--------C     244
PZR61736.1          PDLSDNGMA-----SCEVEGMPVLFLRSPDALYAYRNQCPRCLVGLSRASL--------R     251

WP_011728268.1      NGTLTCPWHGFCYDAT-SGECMSAPGVQLEPLPLRIDDGDIWVRVTG-------   295
WP_013678347.1      AGTLTCPWHGFCYDAT-SGECLSAPGAQLEQLPVRVDGDRIWVRVHT-------   306
WP_053382025.1      DGVITCPYHGFRYDLS-SGECLTAPEVQLVSHAVRVVGSKVEVKLRS-------   294
WP_012963098.1      KGVLVCPWHRFEYSIT-SGECLTVPYVQLVSVPTKIQNGWVLLKVR--------   275
WP_015213936.1      KGIITCSAHEFQYDLK-TGKCLTTPDISLHSYPVKIKGDKVYIKVPNSNQK---   280
WP_015115404.1      NGIITCPVHGFQYQLD-TGQCLT-ADVPLQSYLVQIKGDKVFVKFSR-------   280
```

```
WP_016860574.1      ---------------------------------------------------   158
WP_003894102.1      GQVLRCPHCATGFDVVHAGLSD---SAQLEPVPVLMRDGVPTLALRSTSGGAGP   307
WP_041978571.1      DVMLVCPVCGRRYDVVRAGRASDGANPHLEPLPLLLEHGRAKIALPA-------   285
WP_084922766.1      DAVLRCPTCRTHYDVRRAGIGVEDPDGHLDPLPVLTRDGVLSVAVPAAVTG---   311
WP_010988763.1      ---------------------------------------------------   196
PZR71543.1          EAILTCAACQARFDVKLAGRGVDN-ELHLEPLPLLQTDGEVRLAGAAKS-----   292
PZR61736.1          WPLLACVSCGQEYDVVKAGRAPDQPELHIEPFPLVVSGDKVQLAIPVVA-----   300
```

Figure S6. Conservation of cysteine and histidine residues predicted to bind iron-sulfur clusters in HucE and HhyE proteins. (**a**) WebLogo of the 52 full-length HucE sequences shown in **Figure 1**. (**b**) WebLogo of the 25 full-length HhyL sequences shown in **Figure 1**. The numbering refers to the residues of *M. smegmatis* HucE and HhyL respectively as per **Figure S5**.

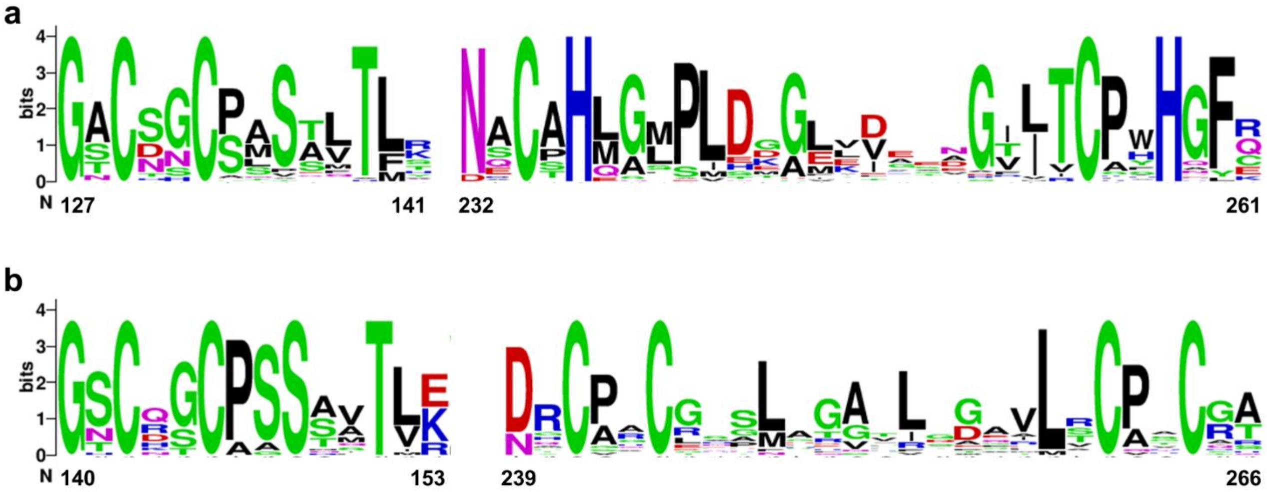

Figure S7. Original native polyacrylamide gels showing staining of hydrogenase activity. (**a**) Huc activity and (**b**) Hhy activity. The left gels show hydrogenase activity stained with the artificial electron acceptor nitroblue tetrazolium in a H_2-rich atmosphere. The right gels show protein marker and total protein stained with Coomassie Blue.

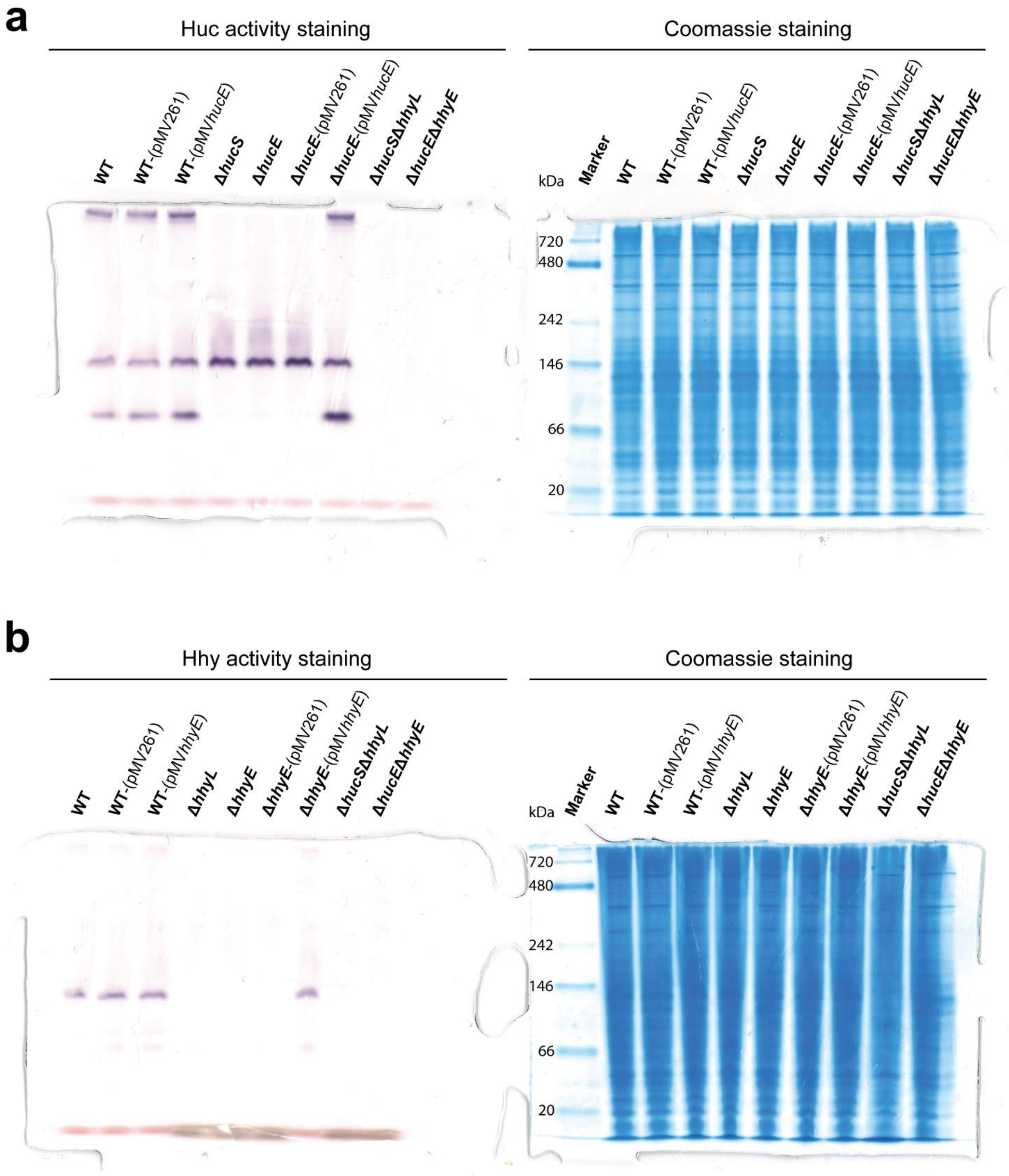

Table S1. Bacterial strains and plasmids used in this study.

Strain	Description	Reference
mc^{2}155	Electrocompetent, wild-type strain of *Mycobacterium smegmatis*	(Snapper et al., 1990)
Δ*hucE*	mc^{2}155 with markerless deletion of MSMEG_2268	This study
Δ*hhyE*	mc^{2}155 with markerless deletion of MSMEG_2718	This study

Δ*hucE*Δ*hhyE*	mc^{2}155 with markerless deletions of MSMEG_2268 and MSMEG_2718	This study
Δ*hucS*	mc^{2}155 with markerless deletion of MSMEG_2262	(Berney et al., 2014b)
Δ*hhyL*	mc^{2}155 with markerless deletion of MSMEG_2719	(Berney and Cook, 2010)
Δ*hucS*Δ*hhyL*	mc^{2}155 with markerless deletions of MSMEG_2262 and MSMEG_2719	(Berney et al., 2014b)
DH5α	Chemically competent, wild-type strain of *Escherichia coli* $F^–$ *endA1 glnV44 thi-1 recA1 relA1 gyrA96 deoR nupG purB20* φ80d*lac*ZΔM15 Δ(*lacZYA-argF*)U169 *hsdR17*($rK^–mK^+$) $λ^–$	Thermo Fisher
TOP10	Chemically competent, wild-type strain of *E. coli* $F^–$ *mcr*A Δ(*mrr-hsd*RMS-*mcr*BC) φ80*lac*ZΔM15 Δ*lac*X74 *rec*A1 *ara*D139 Δ(*ara-leu*)7697 *gal*U *gal*K $λ^–$ *rps*L(Str^R) *end*A1 *nup*G	Thermo Fisher
**Plasmid**	**Description**	**Reference**
pX33	Gm^r, *sacB*, mycobacterial Ts ori, p15A ori, *xylE*	(Gebhard *et al.*, 2006)
pX33-*hucE*	pX33 harbouring the MSMEG_2268 deletion construct	This study
pX33-*hhyE*	pX33 harbouring the MSMEG_2718 deletion construct	This study
pMV261	Kan^r, mycobacterial oriM, pBR322 ori, P_{hsp60} promoter	(Stover et al., 1991)
pMV*hucE*	pMV261 harbouring MSMEG_2268 gene insert	This study
pMV*hhyE*	pMV261 harbouring MSMEG_2718 gene insert	This study

Table S2. Primers used in this study.

Primer	Sequence (5' to 3')	Enzyme
del2268_fwd	aaaACTAGTGTGTCAGGTTCGCATCGAC	SpeI
del2268_rvs	aaaACTAGTTCACAACCGATCACGACCG	SpeI
2267_chrom_fwd	AGACCCTGTGCACGCGATC	
2269_chrom_rvs	CGGCGACCGCGTCGGAC	
del2718_fwd	aaaACTAGTATGACCACCACAGCTCCCAA	SpeI
del2718_rvs	aaaACTAGTTCATGGGATGTCCTCCCGC	SpeI
2717_chrom_fwd	GCAGCGTCCTTCACGGA	
2719_chrom_rvs	GGTAACCCTCGTAGAGCA	
PstI-2268_for	atacatCTGCAGAAATGACCGCGGCGGTCGAG	PstI
HindIII-2268_rev	atacatAAGCTTCTCATCCGGTGACCCGCAC	HindIII
BamHI-2718_for	atacatGGATCCAATGGCGCCCGCCGTCACG	BamHI

HindIII-2718_rev	atacatAAGCTTCTCACGGTCCGGCCCCACC	HindIII

Table S3 (xlsx). Distribution and sequences of HucE and HhyE proteins in microorganisms encoding the group 2a and 1h [NiFe]-hydrogenases.
